# Impact of Coding Curriculum on Dermatology Resident Billing

**DOI:** 10.7759/cureus.24148

**Published:** 2022-04-14

**Authors:** Shayan Owji, Michael Tassavor, Joseph Han, Alexandra Golant, Cula Svidzinski, Jonathan Ungar

**Affiliations:** 1 Dermatology, Icahn School of Medicine at Mount Sinai, New York, USA

**Keywords:** dermatology residency curriculum, residents, revenue, cpt, e/m, residency, coding, billing

## Abstract

Background

Competent medical coding is key to maintaining a successful dermatology practice. Resident billing performance can have significant financial implications for the academic institutions employing them. During their residency training, dermatology residents commonly find themselves responsible for the billing of patient encounters. However, despite the importance of adequate knowledge and skill in medical coding, recent data show inadequacies in this aspect of resident education. The goal of this study is to evaluate the impact of an interventional coding curriculum on dermatology residents’ billing accuracy at our institution.

Methodology

Billing data, including evaluation and management (E/M) level of service, procedural codes, and current procedural terminology modifiers (if applicable) were queried from the electronic medical records (EMR) at a resident clinic seeing patients on three half-days each week. Billing codes were gathered from patient visits occurring in two separate time periods, before and after the intervention. The intervention consisted of monthly resident lectures on E/M and procedural billing in outpatient dermatology with associated quizzes. Billing accuracy was verified by three attending dermatologists through chart review and compared between the two time periods.

Results

Overall, billing data from 532 patient visits, 267 from the pre-intervention period and 265 from the post-intervention period, were checked for accuracy. The accuracy of resident-billed E/M levels of service was similar between the pre- and post-intervention periods (44.3% vs. 44.8%). Similar rates of undercoding and overcoding were noted between the pre- and post-intervention periods (35.2% undercoded and 8% overcoded vs. 35.7% and 8.9%, respectively). However, substantial improvements were noted in the rate of errors with procedural codes and modifiers in the post-intervention period. Overall, 21.9% of procedural codes were incorrectly billed pre-intervention compared to 3.7% post-intervention (p < 0.05). Moreover, 55.2% of modifiers were incorrectly billed pre-intervention versus 27.3% post-intervention (p < 0.05).

Conclusions

Our analysis suggests that billing lectures yielded a clear improvement in resident billing accuracy at our institution. While there was no improvement in E/M coding, there was a significant improvement in the usage of procedural codes and modifiers. Similar analyses can be used by other residency programs to monitor resident billing performance and the efficacy of educational programs on medical billing.

## Introduction

The objective of a residency program is to produce physicians who are ultimately proficient at patient care as well as practice management. In modern medicine, competent medical coding and billing is central to maintaining a successful practice. Therefore, it is key that residents receive ample and effective training on medical coding.

Moreover, residents’ billing performance can have significant financial implications for the academic institutions employing them. During their residency training, dermatology residents commonly find themselves responsible for the billing of patient encounters. This is especially true in outpatient clinics that rely heavily on resident physicians. However, despite the importance of adequate resident knowledge and skill in medical coding, recent data have shown inadequacies in this aspect of resident education. Numerous studies across different specialties have shown that resident education in medical coding is lacking, leaving residents ill-prepared upon entering practice [1-5]. A recent nationwide survey of dermatology residents demonstrated that only 37% of residents felt confident in their billing abilities [6]. This shortage of confidence is not limited to dermatology, with 85% of surgical residents also reporting in a survey that they felt they were novices at coding [7]. Highlighting the financial burdens of improper billing, two studies evaluating the billing performance of internal medicine and family medicine residents revealed that inaccuracies in medical coding resulted in annual losses of roughly $500,000 in each case [8,9].

Patient encounters are billed by applying a complex set of rules to determine current procedural terminology (CPT) codes, sometimes with their appropriate code modifiers when applicable. CPT codes, licensed by the American Medical Association (AMA), are individual five-digit codes representing the various services that can be billed for in healthcare. There are six categories of CPT codes, consisting of evaluation and management (E/M), anesthesia, surgery, radiology, pathology and laboratory, and medicine. E/M and surgery codes, signifying medical decision making (MDM) and procedures, respectively, account for the lion’s share of billed codes in outpatient dermatology.

This study assesses the potential impact that focused educational coding interventions may have on dermatology residents’ billing performance. Our intervention consisted of monthly resident lectures on E/M and procedural billing in outpatient dermatology and associated quizzes, which were subsequently reviewed with residents to maximize retention.

## Materials and methods

This study was conducted in a dermatology residency program at a large, urban academic institution. Billing data were extracted from our institutional electronic medical record (EMR) for visits occurring at a dermatology resident clinic seeing patients three half-days per week. CPT codes corresponding to the E/M level of service and procedures performed, with their associated code modifiers when applicable, were collected from two different time periods: one prior to the start of the intervention and one after. As the educational intervention was implemented in March 2021, January-February 2021 was selected as the pre-intervention period and September 2021 was selected as the post-intervention period. Two months were selected as the pre-intervention period due to significant downbooking during that time caused by the coronavirus disease 2019 pandemic.

Residents’ billing accuracy was verified by three expert dermatologists through chart review. Over the course of four months, the dermatologists were provided datasheets containing visit information for up to 50 resident-billed outpatient visits at a time from the two study periods. Using the provided information to access the visit notes in the EMR, the dermatologists were tasked with inputting the correct E/M code, procedural codes, and applicable modifiers for each visit into the corresponding sections on the datasheet after reviewing each visit note. Resident-billed visits were assigned to each dermatologist in a 1:1 ratio from the pre- and post-intervention periods to ensure potential differences in billing methodologies among dermatologists did not disproportionately impact any one period.

The billing codes inputted by dermatologists were cross-referenced with the resident-billed codes collected from each period, and any billing errors by residents were recorded. The rate of errors in distinctive areas of coding performance, including E/M, procedural, and modifier coding, were determined and compared between the two time periods. Significance testing of differences between pre- and post-intervention error rates was performed using Pearson’s chi-squared test.

## Results

**Figure 1:**
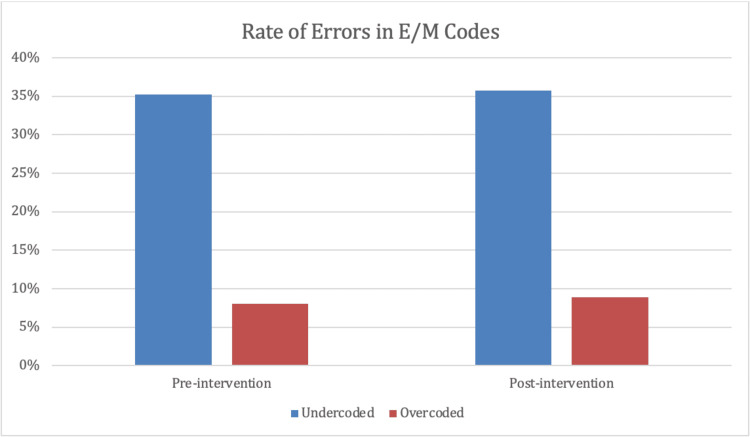
Rate of errors in billed E/M code. Percentage of resident-billed E/M codes billed erroneously, broken down by rates of undercoding and overcoding, during the pre- and post-intervention periods. E/M: evaluation and management

In total, billing data from 532 patient visits, 267 from the pre-intervention period and 265 from the post-intervention period, were evaluated for accuracy. Residents’ accuracy with E/M coding remained nearly unchanged between the pre- and post-intervention periods (44.3% vs. 44.8%, respectively). Further, the rate at which residents undercoded and overcoded E/M levels of service similarly remained unchanged between the pre- and post-intervention periods (35.2% undercoded and 8% overcoded pre-intervention vs. 35.7% and 8.9% respectively post-intervention; Figure 1).

**Figure 2:**
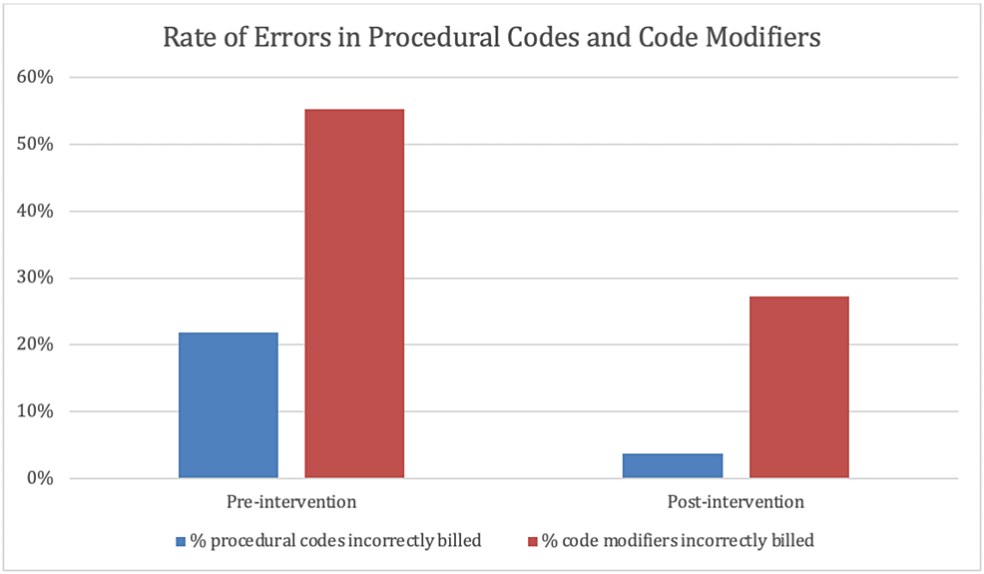
Rate of errors in procedural codes and code modifiers. Percentage of resident-billed procedural codes and code modifiers billed erroneously during the pre- and post-intervention periods.

Significant improvements, however, were seen in the rate of errors with procedural codes and code modifiers after the intervention (Figure 2). Overall, 21.9% of procedural codes were inaccurately billed pre-intervention compared to 3.7% post-intervention (p < 0.05). Moreover, 55.2% of code modifiers were wrongly billed pre-intervention versus 27.3% post-intervention (p < 0.05).

## Discussion

As detailed in prior studies [1,2,4,6,9], medical coding represents an area of resident education that warrants more focused instruction across the spectrum of medical specialties. Deficiencies in resident coding competencies come as no surprise, however, given that frequent changes in coding systems, coding modifiers, and the presence of over 70,000 codes have made medical coding for outpatient visits increasingly complicated [10,11]. Further, there is a dearth of existing research examining medical coding and billing training during residency despite medical coding and billing being designated as one of the six core competencies by the Accreditation Council for Graduate Medical Education (ACGME) [12].

Surveys of dermatology programs have demonstrated an expressed interest by residents in furthering their medical coding and billing knowledge [6]. Given the demonstrated need for increased resident coding competency and a desire to better understand medical coding by residents, it is of benefit to determine the most effective instructive methods for teaching coding in residency curricula. To our knowledge, this is the first study describing the tangible impact of an educational coding curriculum on dermatology residents’ billing performance. As E/M and surgery (procedure) codes comprise the bulk of billing volume in both academic and private dermatology practices, our implemented lectures focused on teaching residents the methodology of applying the complex set of rules necessary to determine applicable CPT codes in these two coding categories.

We found that the intervention greatly improved the billing accuracy of dermatology residents at our institution, causing significant decreases in the rate of errors for resident-billed procedural codes and code modifiers. On the contrary, E/M coding remained essentially unchanged by the intervention. This could be attributed to the relatively greater difficulty of arriving at an accurate E/M code for a visit given the multitude of factors, including the type of history, examination, and MDM, which must be taken into consideration in the billing process. Competence with procedural codes and code modifiers, by virtue of being comparatively more straightforward, appears to be more responsive to instructive efforts. More exhaustive and comprehensive educational approaches are likely needed to perceptibly improve E/M coding. Of note, during both the pre- and post-intervention periods evaluated, the vast majority of residents’ E/M coding errors were attributed to undercoding rather than overcoding of E/M levels of service, which is consistent with prior studies demonstrating residents’ tendency to undercode [13-15]. Though we have no way of ascertaining why visits were more often undercoded, possible reasons include a lack of knowledge, an effort to lessen costs for underinsured or uninsured patients, or a general perception that there is no meaningful benefit to maximizing billing.

While this study suggests lecture-based educational interventions clearly and substantially improve certain areas of billing competency while leaving others unaffected, it has its limitations. The data were collected from only a single institution, and the pre- and post-intervention periods evaluated represent limited time periods. More large-scale and long-term studies may produce varying results.

## Conclusions

Implementation of a lecture-based coding curriculum at our institution significantly improved dermatology residents’ billing accuracy with procedural codes and code modifiers, yet it had no impact on E/M billing. Residents consistently undercoded, rather than overcoded, E/M levels both before and after the implementation of any intervention. Further research on the efficacy of different educational approaches for improving distinct aspects of resident billing performance is necessary to guide the refinement of residency curricula.
